# Animal Models of Neurodegenerative Disease: Recent Advances in Fly Highlight Innovative Approaches to Drug Discovery

**DOI:** 10.3389/fnmol.2022.883358

**Published:** 2022-04-19

**Authors:** Judith A. Tello, Haley E. Williams, Robert M. Eppler, Michelle L. Steinhilb, May Khanna

**Affiliations:** ^1^Department of Pharmacology, College of Medicine, University of Arizona, Tucson, AZ, United States; ^2^Center of Innovation in Brain Science, Tucson, AZ, United States; ^3^Department of Biology, Central Michigan University, Mount Pleasant, MI, United States; ^4^Department of Molecular Pathobiology, New York University, New York, NY, United States

**Keywords:** neurodegenerative diseases, Alzheimer's disease, cell culture models, animal models, Drosophila, drug discovery

## Abstract

Neurodegenerative diseases represent a formidable challenge to global health. As advances in other areas of medicine grant healthy living into later decades of life, aging diseases such as Alzheimer's disease (AD) and other neurodegenerative disorders can diminish the quality of these additional years, owed largely to the lack of efficacious treatments and the absence of durable cures. Alzheimer's disease prevalence is predicted to more than double in the next 30 years, affecting nearly 15 million Americans, with AD-associated costs exceeding $1 billion by 2050. Delaying onset of AD and other neurodegenerative diseases is critical to improving the quality of life for patients and reducing the burden of disease on caregivers and healthcare systems. Significant progress has been made to model disease pathogenesis and identify points of therapeutic intervention. While some researchers have contributed to our understanding of the proteins and pathways that drive biological dysfunction in disease using *in vitro* and *in vivo* models, others have provided mathematical, biophysical, and computational technologies to identify potential therapeutic compounds using *in silico* modeling. The most exciting phase of the drug discovery process is now: by applying a target-directed approach that leverages the strengths of multiple techniques and validates lead hits using Drosophila as an animal model of disease, we are on the fast-track to identifying novel therapeutics to restore health to those impacted by neurodegenerative disease.

## Introduction

Advancements in pharmaceuticals and medical technology have led to fewer deaths from infections and other ailments and consequently, the average life expectancy has increased (Mishra, 2016). It is estimated that by the year 2050, the global number of adults over 80 will be triple that of 2015 (Jaul and Barron, 2017). With increased life expectancy comes a new medical challenge: aging diseases. Aging diseases, or chronic age-related diseases, are a broad category of ailments that emerge in humans as consequence of senescence. As people become older, they may develop chronic age-related diseases, become disabled, and quality of life becomes hindered by their symptoms (Jaul and Barron, 2017; Franceschi et al., 2018). Aging diseases include neurodegenerative diseases, cancer, diabetes, chronic obstructive pulmonary disease, arthritis, and many more (Jaul and Barron, 2017; Franceschi et al., 2018). Neurodegenerative diseases (NDs), such as Alzheimer's disease (AD), Amyotrophic Lateral Sclerosis (ALS), Parkinson's disease (PD), and Huntington's disease (HD) are increasingly becoming the focus of pharmaceutical development because of their rapidly increasing prevalence. In fact, aging is the most significant risk factor for these diseases (Hou et al., 2019). NDs are broadly characterized by progressive loss of neurons ultimately causing behavioral changes, dementia, changes in motor function, and early death (Dugger and Dickson, 2017). We are now becoming acutely aware of the effect that NDs and other aging diseases have on the healthcare system as the population ages. The current medical system is being challenged because effective therapeutics are limited and cures virtually non-existent. There is a significant burden involving the overwhelming task of caring for people affected by these diseases. Faced with the challenge of few effective medical intervention options, this area is ripe for innovation in the discovery of novel therapeutics that can alleviate suffering and extend healthful, disease-free life as well as alleviate the burden on the medical system caused by NDs.

To understand the underlying mechanisms of NDs and to evaluate new therapeutic strategies, different animal models of adult-onset neurodegenerative diseases have been established (Surguchov, 2021). The use of various *in vivo* models, across different species and disease conditions, have provided essential insights into the pathophysiology of NDs, including the characterization of neural cell types, developmental patterns, organ function, and the effects of aging and drugs. While these animal models have proven useful for studying drugs toxicity, sensory function, motor coordination, learning ability, and memory functions, most of them simply fail to recapitulate the complexity of an intact nervous system, and to fully capture the human disease phenotypes. Hence, translation of these findings into the clinic and therapeutic settings have been mostly unsuccessful.

Since the beginning of the 20th century, the tiny fruit fly, *Drosophila melanogaster* has positioned itself as a prominent model organism. Compared to other animal models with sequenced genomes, Drosophila is more cost effective, has a shorter developmental time, and possesses minimal genetic redundancy. Sophisticated genetic tools and the high degree of orthology between fly and mammalian genes make Drosophila a powerful system to study human diseases (Bier, 2005; Pandey and Nichols, 2011). The fly nervous system displays a moderate level of complexity compared to human brain. While Drosophila brain is simpler than mammals, it has well-organized centers for distinct functions such as olfactory, visual, gustatory, motor control, and learning and memory centers (Pereanu et al., 2005). The Drosophila model has made it possible to decipher several pathways responsible for phenotypes reminiscent of human symptoms, such as behavioral and learning deficits, allowing researchers to better understand the etiology of different human conditions and develop new treatments for these disorders in the future.

**Figure 1:**
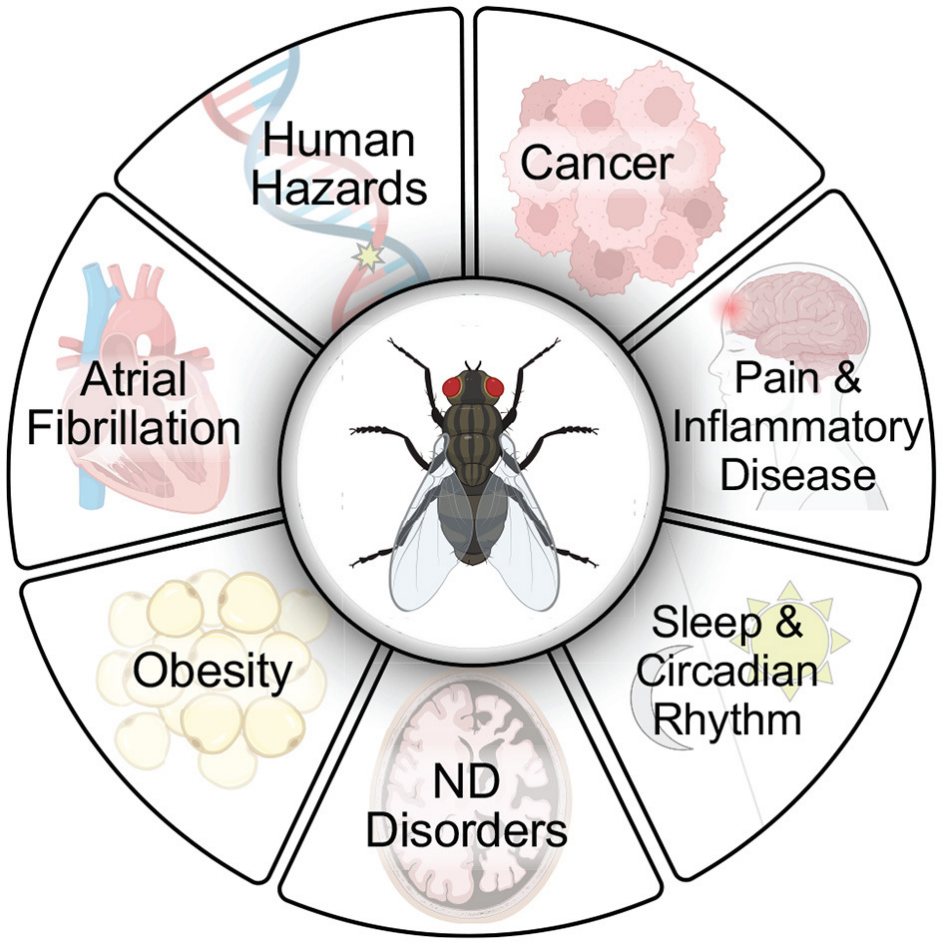
Flies have extraordinary utility for use in drug screening protocols to advance discoveries for human health. Flies have been used extensively in drug discovery, in large part due to their ease of use and relevance to human disease models (Fernández-Hernández et al., 2016; Su, 2019). Small molecules have been identified in screens with relevance to several human disease conditions including cancer (Willoughby et al., 2013; Yadav et al., 2016; Bangi, 2019; Al Outa et al., 2020), pain and inflammatory disease (Grimes et al., 2020), sleep and circadian rhythm (Nall and Sehgal, 2013; Wang et al., 2020), neurodegenerative (ND) disorders including Alzheimer's disease (Hannan et al., 2016; Elovsson et al., 2021), Parkinson's disease (Sanz et al., 2021), Huntington's disease (Sarkar et al., 2007; Schulte et al., 2011), Lysosomal storage disorders (Rigon et al., 2021), Alexander disease (Wang et al., 2016), Friedreich's disease (Seguin et al., 2015), Fragile X syndrome (Chang et al., 2008), Ataxia-telangiectasia (Rimkus and Wassarman, 2018), obesity (Gasque et al., 2013), atrial fibrillation (van Marion et al., 2019), and in human hazard identification (testing potential genotoxins such as food, dietary supplements, cosmetics, drugs, pesticides, herbicides, and nanoparticles) (Barik and Mishra, 2019; Pitchakarn et al., 2021). Created with BioRender.com.

Over the last decades, Drosophila has been instrumental in drug discovery for many facets of human health (Figure 1). These paradigms set the stage for the utility of using flies for gaining a deeper insight into molecular mechanisms that can be leveraged for future research on neurodevelopmental and neurodegenerative diseases. In this review, we will describe NDs and their associated pathogenic proteins, focused mostly in AD. We will discuss the benefits and limitations of cell culture and other animal models to investigate the complex mechanisms of human NDs, culminating with the unequivocal benefits that fly models offer for the future of therapeutics and drug discovery.

## Burden of Neurodegenerative Disorders: Alzheimer's Disease/Dementia

Aging diseases now make up most of the leading causes of death around the world (Heron, 2019). The World Health Organization lists Alzheimer's disease and other dementias as the 7th leading cause of death worldwide and the 2nd leading cause of death in the US and other high-income countries (World Health Organization, 2020). Over the past 40 years, deaths attributed to neurological disorders have increased by 42.1% and years of life lost have increased by 26.2% (GBD 2017 Causes of Death Collaborators, 2018). The Alzheimer's Association reports that between 2000 and 2018, deaths attributed to Alzheimer's Disease increased 146% (Alzheimer's Association, 2020). A decade-long study examining the prevalence of age-related diseases in over 1 million Italian patients over age 65 found that not only did age-related diseases increase in prevalence, but a greater portion also had more than one disease (Atella et al., 2019). In the United States alone, the number of people living with Alzheimer's Disease or other dementia is expected to increase from 56 million in 2020 to 88 million by 2050 (Alzheimer's Association, 2020). The Global Burden of Disease project from the University of Washington Institute for Health Metrics and Evaluation assessed global, regional, and national data on neurological disorders from 1990 to 2016. They found the prevalence of AD and other dementias to increase by 117% while the number of deaths due to AD and other dementias per 100,000 people increased 148% and disability-adjusted life years worsened by 121% (GBD 2016 Neurology Collaborators, 2019).

Current treatments are only palliative—no cures exist for neurodegenerative diseases and the treatments merely mitigate symptoms and delay death (Durães et al., 2018). However, improving quality of life is critical to lessening the burden of NDs. Cholinesterase inhibitors and glutamate regulators are medications that may help lessen or stabilize cognitive symptoms of AD: Donepezil (Aricept®), Rivastigmine (Exelon®), and Galantamine (Razadyne®) are cholinesterase inhibitors prescribed for symptoms related to “memory, thinking, language, judgement, and other thought processes.” These drugs prevent the breakdown of acetylcholine, a neurotransmitter important for memory and learning, thus supporting communication between neurons. Memantine (Namenda®) is a glutamate regulator that supports information processing in the brain and is used “to improve memory, attention, reason, language,” and performing simple tasks. Donepezil and Memantine (Namzaric®), a combination of cholinesterase inhibitor and glutamate regulator, can also be used to treat moderate-to-severe AD. Behavioral and psychological symptoms can also be improved with pharmaceutical treatment after non-drug strategies are attempted. Orexin receptor agonists such as Suvorexant (Belsomra®) treat insomnia in patients living with dementia. Despite providing temporary relief and improved quality of life, the above treatments do not stop the damage to brain tissue caused by AD. Only one drug has been approved for delaying clinical decline: aducanumab (Alzheimer's Association, 2022).

The US Food and Drug Administration (FDA) recently granted accelerated approval of Aduhelm® (aducanumab) for the treatment of AD (FDA, 2021). Despite the controversy surrounding the modest reduction in clinical decline, Aduhelm® was shown to reduce amyloid plaque load and as such, was deemed worthy of approval as a critical new treatment in the fight against AD. Aduhelm® is the first new treatment approved by the FDA in nearly 20 years, highlighting the need for new, effective therapies.

AD and other NDs impact more than just patients with disease. From 2005 to 2015, the estimated cost of AD-associated care expenses rose from $100 billion to $226 billion (Brown et al., 2005; Alzheimer's Association, 2021), with a majority of these expenses mainly from Medicare and Medicaid. Furthermore, these numbers continue to rise as the population ages. In 2015, 5.1% of Americans age 65 and older were living with AD; that number is expected to rise to 13.5% by 2050. Without any change to existing treatments, the number of Americans presenting with clinical AD or mild cognitive impairment due to AD is predicted to increase from 6.08 million in 2017 to 15.0 million by 2060 (Brookmeyer et al., 2018). As a result of the predicted increase in AD prevalence, AD-associated care expenses are expected to approach $1.1 billion by 2050. Delaying onset of AD and other NDs is critical to improving quality of life for patients and reducing the burden of these diseases. The Alzheimer's Association proposed the implementation of a theoretical treatment delaying the onset of AD by 5 years would decrease costs to Medicare, Medicaid, individuals, and other payers by $220 billion in the first few years of implementation (Alzheimer's Association, 2015). An assessment done in 2014 estimated that delaying AD by 5 years would decrease prevalence 41%, decrease AD-associated costs by 40% in 2050, and provide 2.7 additional life years (Zissimopoulos et al., 2014). A subsequent projection found delaying onset of dementia by even just 2 years would increase longevity, reduce years with dementia, and decrease the population of people with dementia age 65 and older by 2.2 million by 2040. This study also examined the theoretical effects of reducing incidence of other comorbidities such as diabetes and hypertension. Surprisingly, reducing incidence of diabetes by 50% or eliminating hypertension at middle and older ages did not have significant benefits to the impact and burden of dementia, underscoring the urgent need for therapeutic intervention for NDs (Zissimopoulos et al., 2018).

## Modeling NDs Cell Culture and Model Organisms

Collectively, NDs generally result from improper protein function and aggregation; although each disease encompasses unique proteins and mechanisms of pathology, these disorders frequently share similarities in general pathological processes such as formation of plaques, disease-associated mutations in proteins, and improper protein localization. Table 1 details the major NDs, implicated proteins, and their pathology.

**Table 1:** Neurodegenerative Diseases (NDs) and associated pathogenic proteins.

**ND**	**Protein**	**Pathology**	**References**
AD	Aβ42	Extracellular plaque formation, neurofibrillary tangles	Ross and Poirier, 2004; Bertram and Tanzi, 2005
AD, ALS, FTD, Pick Disease	MAPT/Tau	Mutations in protein; intracellular neurofibrillary tangles	Ross and Poirier, 2004; Bertram and Tanzi, 2005; Chong et al., 2018
ALS	SOD-1	Mutation in protein, aggregation	Ross and Poirier, 2004; Bertram and Tanzi, 2005
	ALS2	Mutation in protein affecting proper function	Bertram and Tanzi, 2005
ALS/frontotemporal lobar degeneration	TDP-43	Mutation, mislocalization, aggregation	Kwong et al., 2008; Buratti, 2015
PD	alpha-synuclein (*SNCA* or *PARK1*)	Aggregation, Lewy body formation, mutation in protein	Ross and Poirier, 2004; Bertram and Tanzi, 2005
	DJ-1 (*DJ1* or *PARK7*)	Mutation in protein	Ross and Poirier, 2004; Bertram and Tanzi, 2005
	PINK-1 (*PINK1* or *PARK6*)	Mutation in protein	Ross and Poirier, 2004; Bertram and Tanzi, 2005
	Parkin (*PRKN* or *PARK2*)	Mutation in protein causing loss of function	Bertram and Tanzi, 2005
	LRRK-2 or dardarin (*LRRK2* or *PARK8*)	Mutation in protein	Bertram and Tanzi, 2005
HD	Huntingtin	Polyglutamine N-terminus mutation	Ross and Poirier, 2004; Bertram and Tanzi, 2005
Prion diseases	Prion proteins	Intracellular, extracellular amyloid plaques	Ross and Poirier, 2004

*AD, Alzheimer's disease; ALS, amyotrophic lateral sclerosis; FTD, frontotemporal dementia; MAPT, microtubule associated protein tau; SOD-1, superoxide dismutase 1; TDP-43, transactive response (TAR) DNA binding protein 43 kDa; PD, Parkinson's disease; SNCA, synuclein alpha; DJ-1, Protein deglycase; LRRK2, leucine rich repeat kinase 2; HD, Huntington's disease*.

Cell culture models of NDs are critical to better understanding the mechanisms and pathology of disease as well as one of the first steps in therapeutic drug discovery. Slanzi et al. (2020) performed a comprehensive review of cell culture models so that information will not be repeated; here we will provide a brief overview of the use of cell culture models to study NDs for drug discovery. Several cell culture models of NDs exist including immortalized cell lines, induced pluripotent stem cell (iPSC)-derived cell lines, patient-derived iPSCs, and organ-like models (Slanzi et al., 2020). Additionally, different cell types can be used to model different aspects of a ND.

Primary neurons isolated from patients are the gold standard for cell culture studies of NDs and have the potential to overcome many of the disadvantages of other cell types. Acquiring cells directly from patients and establishing their growth in culture ensures that the cells will contain the unique genetic collections of genes that ultimately resulted in the development of disease. Compared to animal models, cell culture of human cell lines removes ambiguity of species-specific effects. However, isolating and culturing some cell types from post-mortem patients is difficult, such as for primary dopaminergic neurons. As an alternative to patient cells, primary dopaminergic neurons are frequently obtained from embryonic murine brain tissue for cell culture studies. While these cells are non-human, they are advantageous because they differentiate rapidly in culture conditions and form synapses and neurites, circumventing the issues of obtaining and culturing primary samples as well as replicating human disease for more reliable use (Slanzi et al., 2020).

Traditionally, cell lines used for high-throughput screening (HTS) for drug discovery are cultured in two-dimensional (2D) monolayer systems. Despite the frequency of use, some evidence suggests that these systems are not replicating the complex conditions of cells in tissues and is thought to be a contributing factor to the high failure rate in drug discovery. To remedy this issue, great efforts are being made to develop and optimize three-dimensional (3D) culture systems that better replicate *in vivo* environments. Multiple approaches exist to create the 3D structure; for example, hydrogels, either biological or synthetic, are used to suspend cells in a matrix rather than letting them settle into a monolayer or physical scaffolds to promote growth along a 3D surface. Organoids, or self-renewing 3D cultures, are being looked to as a major change to drug discovery because they have the potential to better replicate physiological conditions (Langhans, 2018). Currently, 2D culture techniques are more commonly used to study NDs and screen therapeutics for those diseases (Korhonen et al., 2018). One study using AD patient-derived 2D cultures found treatment with β-secretase inhibitors showed significant reduction in hyperphosphorylated tau and active glycogen synthase kinase-3β in neurons (Israel et al., 2012). 3D culture techniques for drug discovery are being optimized but are not yet extensively used for initial screening (Korhonen et al., 2018). However, 3D cultures of iPSC-derived cortical neurons have been used for HTS against tau aggregation, so the future use of 3D models for drug discovery is just around the corner (Medda et al., 2016).

Cell culture models of AD have contributed to understanding key physiological mechanisms of the disease, but are not able to replicate the disease in entirety because of the complexities of pathogenesis. Animal-derived cell models of AD fail to replicate the important and inter-connected pathological mechanisms seen in human patients. Most *in vitro* studies of AD have been conducted using patient-derived iPSCs (Penney et al., 2020) and other human tissues as well as CRISPR/Cas9 technology to generate knock-in mutations in cultured human neurons. However, 3D cultures methods seem to be a way to overcome the complications of 2D *in vitro* models of AD. New 3D culture techniques such as organoids are more recent avenues for *in vitro* AD modeling. Creation of “mini-brains” using multiple cell types shows promise for the future of AD studies. Coculturing has allowed for the creation of a perfusion system that mimics the cortical vasculature and in the near future, potential to model more complex features of the brain such as the permeability of the blood-brain barrier (Slanzi et al., 2020). The lack of a representative blood brain barrier (BBB) is a notable disadvantage when modeling NDs *in vitro*: the BBB is the most significant obstacle to effective CNS drug delivery. The BBB can be impacted by NDs and limits what compounds hold therapeutic potential (Slanzi et al., 2020). A compound directly administered to cells in culture may prove promising, but if the compound cannot cross the BBB, it will not reach the affected areas and thus have no therapeutic value. Indeed, previous work has shown that human brain microvascular endothelial cells grown in culture do not maintain their full BBB properties (see Helms et al., 2016, for a detailed review).

Genetic model organisms are easy to maintain in a lab setting, have short lifespan and generation cycles, have the capacity to generate and study mutations, and importantly, address many of the deficiencies of cell culture. Yeast, for instance, is an important organism to study to understand basic molecular processes in eukaryotic organisms. The first complete DNA sequence of a eukaryotic genome was *S. cerevisiae*, which is among the best-studied experimental organisms (Engel et al., 2014). *Saccharomyces cerevisiae* and humans were found to share about 23% homologous genes and there is a high degree of conservation of gene function across shared genes (Botstein et al., 1997; Liu et al., 2017). Although yeast are more simple organisms, their contribution to biological research has been widespread. As a disease model, yeast have been integral for understanding disease-causing genes. Disease-associated genes with unknown function are easily transfected into yeast to elucidate the cellular function. Yeast models are also well-suited for target-based HTS for drug discovery (Denny, 2018). In the context of NDs, yeast have been particularly useful in understanding protein misfolding and subsequent cellular toxicity in HD (huntingtin), PD (α-synuclein), and AD (tau, Aβ), as well as fundamental cellular processes because of the strong conservation of gene function between humans and yeast (Winderickx et al., 2008; Liu et al., 2017; Delenclos et al., 2019). Yeast models have also been effective for studying ALS, oxidative stress, autophagy, and prion diseases (Breitenbach, 2013; Di Gregorio and Duennwald, 2018; Chernoff et al., 2020; Gross and Graef, 2020). Despite all the benefits of yeast models, there are a number of limitations. Yeast are single-celled organisms and lack tissue organization, thus translating effects observed in yeast to multicellular organisms is difficult. Additionally, yeast lack an intercellular inflammatory signaling system that is integral to the pathology of many human diseases, especially NDs (Bilinski et al., 2017).

Animal models are particularly valuable for screening therapeutic compounds since effects can be assessed at the cell, tissue, organ, and system level in a relatively short time frame. Organized tissues and organ systems allow researchers to study the physiological effect of drug delivery on digestion, respiration, circulation, excretion, reproduction, and most importantly, the nervous system. There are advantages and disadvantages to using a particular animal model and translatability to human patients can be highly variable depending on the model used (Dawson et al., 2018).

*Caenorhabditis elegans*, small nematodes, have surprisingly high similarity to humans sharing at least 83% homology with human genes (Lai et al., 2000; Kim et al., 2018). They are also well-suited for genetic manipulation, use of fluorescent technologies, *in vivo* imaging, and are frequently used for modeling human diseases and studying disease pathogenesis (Li and Le, 2013). Additionally, the neuronal signaling conservation makes nematodes good models for understanding underlying mechanisms of neurodegeneration such as genetic interactions and molecular pathways in AD, PD, ALS, HD, and other NDs (Li and Le, 2013). The nervous system of C. elegans is composed of only 302 neurons that make up ganglia in the head and tail and “a spinal cord-like ventral nerve cord” but has sophisticated information processing systems and complex wiring. Despite the small number of neurons, there are 118 different neuron types each expressing many different neurotransmitter receptors and neuropeptides, making them especially useful to study nervous system development (Hobert, 2010). Additionally, the combination of genetic tractability, cost-effectiveness and relatively short lifespan make *C. elegans* a highly attractive model for studying the effect of organismal age on protein aggregation at a molecular level. Another major advantage of using *C. elegans* models is the ability to perform unbiased genetic screens particularly for identifying disease mechanisms in AD and other NDs that are explained by multiple hypotheses (Link, 2006). For example, two new factors involved in tau pathology were identified: SUT-1 and SUT-2 (Alexander et al., 2014). These models can, however, be limited by the ability to replicate the pathophysiology of human NDs. The defined tissue and organ systems of the central nervous system (CNS) and brain that are severely impacted by NDs are not present in the worms. These organisms also do not have adaptive immune systems, so they are not able to replicate inflammation seen in some NDs. Additionally, their small size necessitates using large numbers of worms for sufficient amounts of material for biochemical analyses (Li and Le, 2013; Van Pelt and Truttmann, 2020).

Zebrafish (Danio rerio) models are emerging as viable model organisms to study ND and for drug discovery efforts. Zebrafish genome sequences share about 70% homology with human sequences, and 84% of human disease-associated genes have zebrafish counterparts (Kalueff et al., 2014; Saleem and Kannan, 2018). Zebrafish have the distinct advantage of being a vertebrate model system, and compared to other vertebrate models, have much simpler care requirements and natural habitat, making them more cost effective to maintain with a relatively short generation time. Additionally, they use external fertilization to produce large numbers of offspring, ideal for higher throughput analysis, and the embryos and larvae have high optical clarity, making them well-suited for non-invasive imaging and aids in genetic manipulation. Zebrafish models of AD show neuroanatomical, behavioral, and pathophysiological similarities to human AD patients and are being used as both neuropharmacological and neurogenic models of AD (Saleem and Kannan, 2018). Since compounds are usually added to the water and absorbed randomly through the gills and skin, quantitation is difficult to measure in fish. Additionally, zebrafish can regenerate neurons in certain parts of the brain while mammals cannot, complicating the study of neurodegeneration in the fish (Saleem and Kannan, 2018). Despite these complications, a transgenic zebrafish model expressing tau presents key pathological features of tauopathies and tau-dependent neuronal loss (Paquet et al., 2010). Injection of Aβ42 into the brains of zebrafish embryos resulted in AD-like symptoms: memory loss, cognitive deficits, increased tau phosphorylation (Nery et al., 2014). Increases in okadaic acid concentration leads to an increase in Aβ-plaques and tau phosphorylation and learning and memory deficits (Nada et al., 2016).

Mice and rats are some of the most used animal models for screening therapeutics and other pharmacological compounds because they are more closely related to humans than other models. Mice and humans diverged from a common ancestor about 90 million years ago (Monaco et al., 2015). On the genomic level, mouse and human genomes are about 85% identical and between mice, rats, and humans, the three species have about 95% of genes in common (Bryda, 2013; Uhl and Warner, 2015). Compared to the aforementioned animal models, murine nervous systems possess higher complexity and are more similar to that of humans, allowing for greater translational application (Jucker, 2010). An in-depth analysis of human and mouse brain transcriptome networks revealed gene expression and connectivity were significantly conserved as well as significant preservation of network modules (Miller et al., 2010). Brains in both humans and mice share many developmental milestones on a structural and cellular level, albeit along different timelines. As they develop, humans and mice also share many age-dependent behavioral phenotypes, such as increased socialization, development of working memory, and risk-taking behaviors (Semple et al., 2013). Despite their many advantages, murine models have drawbacks as well. Some RNA binding sites involved in ND pathology are not well-conserved between rodents and humans (Dawson et al., 2018). The lifespan and size of rodents makes them more expensive to rear and cross and limits the scope of high throughput studies. Rodents can live for years, significantly slowing data acquisition and analysis, especially in the context of age-related diseases where disease phenotypes do not appear until the animals are aged significantly (Pandey and Nichols, 2011). While some models effectively mirror human disease pathogenesis, in some mouse models, certain aspects of disease are not well-replicated. Some APP-transgenic mouse models such as the PDAPP and Tg2576 lines show modest neuronal loss confined mainly to the hippocampus (Duyckaerts et al., 2008). In human AD patients, neuronal loss is more severe across regions of the hippocampus, the entorhinal cortex, and neocortex (Gómez-Isla et al., 1996; Niikura et al., 2006). PD mouse models that overexpress human wild-type and mutant α-synuclein exhibit development of Lewy bodies and show progressive age-dependent neuropathology and cognitive and locomotor dysfunctions but loss of dopaminergic neurons in the substantia nigra characteristic of PD is not replicated (Jucker, 2010). A major hurdle in the development of therapeutics is the issue of translatability from rodent models to humans. Many therapeutics show promising results in rodent models, but the therapeutic value frequently does not translate to human patients in clinical trials (Franco and Cedazo-Minguez, 2014).

Non-human primate (NHP) models of NDs are likely the closest animal model to study human disease. Humans and chimpanzees diverged from a common ancestor 5–12 million years ago and NHP nervous systems are the closest replication of human physiology (Rogers and Gibbs, 2014; Li H.-W.et al., 2019). Both spontaneous and artificially-induced NHP models of NDs exist including AD, HD and PD (Emborg, 2017; Li H.-W.et al., 2019). Aged NHPs can spontaneously develop characteristics similar to AD patients: Aβ plaque-like structures, neuropathy, and cognitive behavioral changes. However, there are very few sources of aged NHPs. Combined with the long lifespan of these animals, using aged NHP models for studying spontaneous NDs is very expensive, although not necessarily prohibitively, making use of NHPs uncommon. The animals need to be cared for throughout their lives until they are considered “aged” which can be 5–24 years depending on the NHP model used (Dawson et al., 2018; Li H.-W.et al., 2019). Artificially-induced models employ methods such as seeding AD brain lysate into the primate brain, inhibition of cholinergic receptors, formaldehyde damage to neurons through methanol ingestion, and intracerebroventricular injection of streptozotocin (Dawson et al., 2018; Li H.-W.et al., 2019). Artificially induced NHP models are limited by side effects of the agents used to induce damage possibly affecting behavior, potential over-simplification of disease pathology, and requiring invasive surgical procedures as part of the induction process causing harm to the animals (Li H.-W.et al., 2019). Transgenic NHP models are thought to be the best path for finding a cure, but the creation of these models is extremely difficult. The gene transfer method has very low efficiency, necessitating the development of a better method for creating transgenic models for biomedical research. NHP models of PD are being used to study and optimize stem-cell therapies as a dopamine replacement approach and have even identified a novel therapeutic, SK609, that shows reduced cognitive and motor errors as well as improved object retrieval performance in a macaque model of PD (Chen and Niu, 2019; Schneider et al., 2021).

## Flies as a Powerful Alternative

Creating animal models for diseases is not only important for understanding underlying pathological mechanisms of disease but also identifying and preclinical screening of new pharmacotherapeutics to treat NDs. In their review on translatability of findings from Drosophila models to NDs, Shulman et al. (2003) suggested the use of Drosophila as a key tool for the development of novel ND therapeutics (Shulman et al., 2003). Now, almost 20 years later, Drosophila models are being used for just that.

Fruit flies (*Drosophila melanogaster*) are an invertebrate model organism used to model numerous human diseases (Sarkar et al., 2007; Chang et al., 2008; Schulte et al., 2011; Willoughby et al., 2013; Hannan et al., 2016; Wang et al., 2016; Yadav et al., 2016; Rimkus and Wassarman, 2018; Bangi, 2019; Al Outa et al., 2020; Elovsson et al., 2021; Rigon et al., 2021; Sanz et al., 2021). *Drosophila melanogaster* have been thoroughly studied and their genome has been sequenced and annotated. Fruit flies and humans diverged over 700 million years ago (Shih et al., 2015). Despite the time of divergence, the genome of the fruit fly is about 60% homologous to the human genome, and ~75% of human disease-related genes have homologs in *D. melanogaster* (Reiter et al., 2001; Bier, 2005; Lenz et al., 2013; Mirzoyan et al., 2019). While more distantly related to humans than rodents and non-human primates, fruit flies have many advantages as a model especially for use in drug discovery for ND therapeutics (Pandey and Nichols, 2011). One important aspect of modeling NDs is using aged animals. Mentioned earlier, animal models such as NHPs and rodents require significant investment of time and resources to care for the animals until they are considered aged. Fruit flies, however, reproduce much faster than rodents and have larger numbers of progeny, making them well-suited for HTS. The fruit fly has a lifespan of only 2 months, such that aging studies can be accomplished on the scale of weeks or months rather than years (murine models) or decades (NHP models). In some cases, a complex cross scheme is needed to create organisms modeling more complex features of a disease, but the generation time allows these more complex cross schemes to be performed and maintained easily (Pandey and Nichols, 2011; Warr et al., 2018).

There are many structural and physiological similarities between human and *D. melanogaster* nervous systems. Both are mainly composed of neurons and glial cells and they both share neurotransmitter systems, and are organized in similar ways (Leyssen and Hassan, 2007; Freeman, 2015; McGurk et al., 2015). The adult nervous system in Drosophila is divided into two main parts: the central nervous system (CNS) and the ventral nerve cord (VNC). In larvae, the brain is divided into two hemispheres that develop into the adult CNS and eyes, and a subesophageal ganglion or ventral ganglion that develops into the adult VNC (Jeibmann and Paulus, 2009). Mushroom bodies (MB), paired neurophil structures in the CNS, are involved in processing sensory information and are generally considered the memory centers of the fly brain (Campbell and Turner, 2010). MB contain neurons called Kenyon cells (KCs). Across most insects including Drosophila, KCs receive visual, gustatory, olfactory, and mechanosensory information as well as learning and memory related to olfactory stimuli (Akalal et al., 2006; Stopfer, 2014). Aside from the functions of KCs, the MB are involved in sleep regulation, information transfer, and formation and retrieval of memories (Perez-Orive et al., 2002; Joiner et al., 2006). The insect MB has been compared to three mammalian brain regions: (1) the hippocampus, based on the shared involvement in learning and memory; (2) the cerebellum, due to the roles in learning, precise motor movements, and densely packed tracts of axons; and (3) the piriform cortex, because both structures are closely linked to the olfactory system sensory layer. The compound eye is also an integral part of the CNS. The Drosophila eye is made up of ~800 ommatidia, optical units that include eight photoreceptor cells, four cone cells, and two primary pigment cells, surrounded by additional support cells (mainly bristles and other pigment cells) (Mollereau and Domingos, 2005). The adult eye develops from the eye-antennal imaginal disk structure in larva (Cutler et al., 2015). Because the structure of the adult eye is highly ordered and repetitive, any slight abnormalities are easily observed (Baker et al., 2014) and is useful in identifying and studying toxic proteins using the rough eye phenotype, discussed later.

The VNC is located posterior to the brain and connects to the CNS through the neck. Composed of a single consolidated ganglion, the VNC is the main area for reception and integration of sensory information as well as generating the actions underlying most behaviors such as walking, grooming, jumping, flying, courtship, and copulation (Court et al., 2020). The VNC is analogous to the vertebrate spinal cord; clusters of neurons are grouped together by the neuroblast progenitor cell from which they arose and have characteristic molecular patterns associated with a cluster's location (Harris et al., 2015).

One important feature of fly models is the presence of a blood brain barrier (BBB) in the Drosophila CNS. A comprehensive review of the Drosophila BBB can be found at Limmer et al. (2014). Having a structure to separate the CNS from the rest of the body provides direct support and protection of the nervous system and maintains metabolic and ionic balance in the different environments. Vertebrate systems are highly vascularized, and blood is circulated by the heart, whereas insect systems are bathed in hemolymph, a blood-like solution that lacks the presence of oxygen-transporting blood cells, circulated by a heart-like structure. In higher order vertebrates the BBB is formed primarily by the brain vascular endothelium, whereas in insects the BBB is formed by glial cells. Though the gross anatomy of the BBB is different between vertebrates and invertebrates, morphological similarities are found. Mammalian BBBs are formed by a monolayer of tightly connected vascular endothelial cells covered by pericytes, further surrounded by astrocytic glia endfeet. Polarized endothelial cells and pericytes lead to the formation of tight junctions that control exchange of solutes between the fluid in the CNS and the blood. The invertebrate BBB is also a compound structure, composed of two surface glia cell types, and a basement membrane known as the neural lamella. Both the vertebrate and invertebrate barriers express tight/septate junction and adherens junction proteins, as well as various xenobiotic ATP-binding cassette and solute carrier transporters required to maintain CNS homeostasis (DeSalvo et al., 2014; Limmer et al., 2014). Similarly, each cellular component in the BBB of all animals appears to have a coordinated role in maintaining BBB integrity and for signaling purposes. In the context of drug discovery for NDs, BBB permeability is key for compounds targeted to the CNS. Compounds that cannot cross the BBB will not reach target sites necessary to elicit a therapeutic effect. Although structurally mammalian and Drosophila BBBs are similar, there may be blood-brain permeability differences. DeSalvo et al. (2011) developed an approach to perform live imaging as a screening methodology for physiologic and anatomic characterization of the Drosophila BBB. This approach involved visualizing a change in the fluorescence intensity in the fly eye after injecting a chemical fluorophore into the Drosophila brain. DeSalvo et al. (2011) argued that the different intensities were due to a change in permeability of the blood-eye barrier, and that this approach can be used to screen for genetic modifiers of BBB integrity as well as identify candidate genes expressed in different layers of the compound BBB. There is still much to learn about the differences in the BBB between fly and human, how these barriers are regulated, and how the individual cells in each specimen respond to pathological perturbations. Despite this potential for limited translatability, initial screening of a compound in a Drosophila model of an ND can provide key early insights into permeability properties translatable to human systems.

Both human and fruit fly nervous systems have neurons and glial cells that operate on the same principles and use many of the regular canon of neurotransmitters to communicate: dopamine, acetylcholine (Ach), glutamate, and GABA neurotransmitter systems are conserved. Although there are conserved similarities between neurotransmitter systems in vertebrates and invertebrates, there are also key differences. For example, the fly contains no adrenergic system, instead they possess their own norepinephrine equivalent, octopamine (although their physiological functions are similar in both systems). Furthermore, in mammals, the primary excitatory neurotransmitter in the brain is glutamate, and at the neuromuscular junction (NMJ), it is Ach; conversely, flies use glutamate at the NMJ, and ACh in the brain (see Nichols, 2006, for a detailed review). Interestingly, while glutamate is one of the best characterized neurotransmitters in the mammalian CNS, in flies it remains one of the least understood.

In the context of NDs, *D. melanogaster* models have been used to discover novel mechanisms of pathogenesis thanks to the high degree of conservation of molecular pathways between humans and flies (Shulman et al., 2003; Lenz et al., 2013). The basic principles of neurocircuitry are conserved at the molecular, developmental, and functional level between flies and humans (McGurk et al., 2015). Additionally, many complex behaviors are shared between the humans and flies that are especially relevant to human neuropsychiatric disorders such as sleep, memory, and aberrant aggression (Pandey and Nichols, 2011). Beginning with development of the embryonic nervous system, CNS cell fate can be affected by mutations in functionally orthologous genes shared between flies and humans (Nichols, 2006). The histology of Drosophila CNS is also highly complex. Between Drosophila glial cell types exist overlapping functions involved in neural circuit formation, function, plasticity, and pathology. Three major neuron-associated glial cell types are present in Drosophila nervous system: (CNS)-astrocytes, cortex glia and ensheathing glia. These cells act similarly to mammalian astrocytes to regulate many processes affecting brain structure, neural circuit remodeling, neural circuit function and behavior, and ensheathing, among others (Freeman, 2015). In addition to structural and functional similarities, a number of conserved behaviors exist between flies and mammals that are not conserved between mammals and other simpler model organisms including circadian rhythm, sleep, learning and memory, courtship, feeding, aggression, grooming, and flight navigation (Nichols, 2006).

Another major advantage of fruit fly models is the ease of genetic manipulation and potential for forward and reverse genetic screens (Singhal and Jaiswal, 2018). Many systems can be used in *D. melanogaster* to regulate existing gene expression or create transgenic lines to produce a desired disease model (Deal and Yamamoto, 2018). The yeast FLP (flippase recombinase)/FRT (FLP recombination target) system (FLP/FRT) system can be used in Drosophila to manipulate gene expression (Frickenhaus et al., 2015). The FLP/FRT system uses a mutation of interest and a scorable marker to create mutant clones (Deal and Yamamoto, 2018). The system also has multiple applications such as the creation of overexpression clones, inducing whole mutant tissues, and conducting RNAi screens (Weasner et al., 2017).

Other systems, including GAL4-UAS, the LexA-lexAop, and the QF-QUAS are all binary systems that use promoter binding to affect gene expression, with the GAL4-UAS system being the most widely used to create many *D. melanogaster* models of ND (del Valle Rodríguez et al., 2011). The bipartite system utilizes both a GAL4 driver that expresses the yeast transcription factor GAL4 in a tissue specific pattern, and Upstream Activation Sequences (UAS) that include binding sites for GAL4 to induce gene expression. Gene expression in progeny involves a cross between two lines: (1) a fly in which the transgene of interest is inserted downstream of a UAS; and (2) a fly in which GAL4 is downstream of a site-specific promoter gene. In progeny, the GAL4 transcription factor is expressed and binds to the UAS sequences triggering expression of the transgene in the desired location. This system allows for great control of gene expression by selecting appropriate GAL4 drivers and UAS-linked transgenes. Ongoing work seeks to expand the use of the GAL4-UAS system through the development of inducible models, such as the GAL4 GeneSwich adaptation or hormone inducible GAL4 systems (Jackson, 2008; Jones, 2009; Gama Sosa et al., 2012; Jenett et al., 2012; McGurk et al., 2015).

Loss of function (LOF) approaches serve to identify the contribution of genes in a particular biological context in the fly, with the goal of translating these results to human orthologs. Traditionally, these approaches require the use of chemical mutagens such as ethyl methanesulfonate (EMS) or radiation such as X-rays to introduce random mutations throughout the genome in the whole animal, but are very time consuming, have low through-put, and are difficult to apply to age-dependent phenotypes (Deal and Yamamoto, 2018). Certain genes are essential for nervous system development early in the fly, therefore knocking them out completely could cause early lethality and these genes could be missed in genetic screens. RNA interference (RNAi) works around this issue by knocking down gene expression on the RNA level in a tissue-specific manner. RNAi has been used in this way to identify genes with neuroprotective functions such as Presenilin and Nicastrin subunits of γ-secretase (Deal and Yamamoto, 2018). The selective targeting of a specific gene or genes for knockdown using RNAi also resolves the above issues with using mutagens like EMS (Deal and Yamamoto, 2018). Described earlier, the FLP/FRT system can also be used to create LOF mosaic clones (Weasner et al., 2017). Another major type of LOF screen involves the integration of transposable P elements that cause chromosomal deletions of genes flanking the inserted P-element. P-elements have also been utilized to create new GAL4 expression lines with cell-type specificity (Rigon et al., 2021).

In contrast, gain-of-function (GOF) approaches often involve transgenic flies expressing human DNA to identify genes, proteins, and pathways involved in genetic suppression or enhancement. Use of genetic systems, such as the GAL4-UAS system, is integral to GOF approaches. By studying the effects of toxic genes in fly models, new targets for therapeutics can be uncovered. Upon gene expression using one of the above genetic systems, molecular pathways affected by the gene can be evaluated. For example, Casas-Tinto et al. (2011) targeted human amyloid-β 1–42 (Aβ42) expression to the eye and observed disruption of the eye structure as a result of Aβ toxicity. Further investigation found that Aβ activates the expression of ER stress response factor XBP1s, a protein that showed neuroprotective potential. By studying this pathway, XBP1s and ryanodine receptor RyR were identified as novel targets for AD therapeutics (Casas-Tinto et al., 2011). Using GOF approaches, newer and better Drosophila models can be created that more closely recapitulate the features of human disease, paving the way for drug discovery largely via screening therapeutic compounds (screening methodologies are discussed in detail below). A comprehensive review of *D. melanogaster* models of neurodegenerative diseases can be found by Bolus et al.; here we will focus on the application of fly models of ND to identify compounds with translational potential for human therapeutic applications (Bolus et al., 2020).

As summarized in Table 2, several laboratories have effectively used fly models of the major neurodegenerative diseases to test the effect of specific candidate compounds or screen compound libraries. In the context of aging diseases, because of their short lifespan, and low-cost care requirements, flies are advantageous for HTS. Flies only live about 50–80 days so while mice can live up to 3 years, using a fly model for survival studies speeds up screening (Coleman et al., 1966; Bauer et al., 2004; Linford et al., 2013). Additionally, the similarities in nervous system function and organization allow for *in vivo* exploration of nerve function. In the context of drug discovery, fly models pose a unique advantage for the initial discovery process. Even though the throughput using flies is lower than using mammalian cell culture for screening for positive hits from a compound library, *D. melanogaster* systems can identify higher quality hits from fewer screened compounds. Screening in a whole organism allows for elimination of unpredicted toxicity when translating hits from mammalian cell culture to rodent models (Pandey and Nichols, 2011).

**Table 2:** *D. melanogaster* models of ND used for small molecule screening.

**Drosophila model**	**Dev. stage**	**Screen design**	**Output measurement**	**References**
A53T/A30P α-synuclein mutants (PD)	Adult	Candidate drug Decalepis Hamiltonii extract (known antioxidant) was fed to the flies for 21 Days.	Negative Geotaxis Assay PQ Toxicity assay Lifespan assay	Jahromi et al., 2015
Lrrk2 mutants (PD)	Adult	Piceatannol, Thymoquinone, and Esculetin mixed with DMSO was incorporated into the fly food and fed to mutants.	Oxidative state assays Climbing assays Immunostaining Western blots	Angeles et al., 2016
PINK1 mutants (PD)	Larval	High-throughput screening of a compound library identified 2 small molecules (T0466 and T0467) which were effective.	Immunostaining Western Blots	Shiba-Fukushima et al., 2020
PINK1 mutants (PD)	Larval	High-throughput screening of 320 compounds which found 3 effective compounds (MRS1220, tranylcypromine, bromocriptine).	Immunostaining Crawling assays	Yamaguchi et al., 2020
DJ-1 double knockout mutants (PD)	Adult	Exposed mutants to a variety of oxidative stressors (paraquat), proteasome blockers (MG132), and agents that unfold proteins (dithiothreitol).	Lifespan assay Western blots	Meulener et al., 2005
DJ-1 mutants (PD)	Adult	Screened a variety of human used drugs for PD and other experimental drugs at highest possible concentration. All drugs were fed to flies.	Climbing assay Peroxide measurement Carbonyl formation assessment	Sanz et al., 2017
Expression of human N370S GBA (PD)	Adult	The compound Nicotinamide Riboside was identified for study, and mixed with adult mutant fly food.	Anti -TH staining Climbing assay Dopaminergic Neuron counting	Schöndorf et al., 2018
Aβ42 mutants (AD)	Adult	Extracts from brown alga and prickly pear plants were fed to flies.	Lifespan assay Climbing assay Western blots Liposome assay	Briffa et al., 2017
Aβ42 mutants (AD)	Adult	Extracts from multiple plants were used in a high throughput screen with adult flies.	Protein quantitation Memory tests	Ma et al., 2017
Expression of human APP and BACE1 (AD)	Adult	Flies were fed food mixed with γ-secretase transition state inhibitor L-685,458 throughout their entire lifespan	Lifespan assay Climbing assay Immunostaining for amyloid deposition Courtship Behavior Assay	Chakraborty et al., 2011
Expression of mutations in human tau transgene (tau^R406W^) (AD)	Adult	Biotin was identified as a potential therapeutic and therefore flies were fed various diets containing various levels of biotin.	Locomotor assay Enzyme linked immunosorbent assay Western blotting	Lohr et al., 2020
Expression of mutations in human tau transgene (tau^R406W^ and tau^P301L^) (AD)	Adult	A screen of compounds was conducted that identified Ro 31-8220 as a potential effective treatment. The compound was then mixed with standard fly food.	Lifespan assay Negative geotaxis assay Learning and memory assay	Shim et al., 2019
SOD1 mutants (ALS)	Adult	α-Lipoic acid mixture was mixed into the food and fed to the mutant flies.	Behavior assay Enzyme activity assay Lifespan assay	Wang et al., 2018
Wild Type TDP 43 mutants (ALS)	Larval	A virtual and physical screen of compounds was performed to select for compatible TDP-43 targets. The compatible compounds were then fed to larvae.	Larval turning assay	François-Moutal et al., 2019
Expression of Human Htt at various Poly-Q lengths including Q15 and Q138 (HD)	Cell culture Adult	Screen of a compound library that produced multiple novel drug hits.	Negative geotaxis assay Confocal microscopy	Schulte et al., 2011
Expression of human mutant Htt Exon One (Q93) (HD)	Adult	Screened small molecule library for effectiveness in reducing HD phenotype. Drugs were delivered via mixture with food.	Neurodegeneration assay	Desai et al., 2006
Expression of human mutant Htt Exon One (Q93) (HD)	Adult	Connectivity mapping revealed potential therapeutic genes and chemicals. These identified chemicals were then mixed with food and introduced to the mutants.	Pseudopupil assay	Smalley et al., 2016
Expression of human mutant Htt (Q93) (HD)	Adult Larval	The drug resveratrol was identified to attempt to inhibit Sir2 and Rpd3 genes which play important roles in HDAC activity. Drugs were delivered via a mixture of fly food.	Lifespan assay Neuronal survival frequency	Pallos et al., 2008
Expression of human mutant Htt (Q93) (HD)	Adult	High-throughput screening combined with a FRET assay identified one lead therapeutic compound Y-27632 which was fed to flies.	Rhabdomeres assay	Pollitt et al., 2003
Expression of full length Htt with 16Q and 128Q (HD)	Adult	High-throughput screening identified compounds of interest for study. These were then fed to flies in a mixture of fly food.	Western Blot Filter trap assay HTRF assay c-Raf kinase assay Pull-down assay Autophagy assay Behavioral assay Lifespan assay	Li Z. et al., 2019

As with the previously discussed animal models, there are also limitations with *D. melanogaster* models. An obvious limitation of fly models is uncertainty about important pathogenetic factors that may be vertebrate-specific and thus fail to be replicated in invertebrate models. Structural and functional differences must be considered when using Drosophila models; for example, flies have no substantia nigra, therefore screening compounds that affect this area of the brain in relation to PD is not appropriate. Fortunately, other areas of the brain and genes related to PD can be studied in flies (Pandey and Nichols, 2011). With respect to heart disease, Drosophila has emerged as a useful model to study heart development and model certain aspects of cardiovascular disease, however a greater understanding of the genes and pathways involved in adult human heart disease remain obscure, due largely to important anatomical and physiological differences. *Drosophila melanogaster* have an open circulatory system, with a simple tube-like heart that pumps the hemolymph from the posterior body region toward the anterior, and oxygen is delivered by an independent tracheal system. Vertebrates, by contrast, have an extensive vascular network that facilitates blood delivery throughout the body (Choma et al., 2011). *Drosophila melanogaster* also have a less complex immune system than mammals, making difficult to evaluate the inflammatory response associated with NDs. Differences in pharmacokinetics and pharmacodynamics between humans and flies are also a major hurdle: small molecules may produce significant discrepancies in drug concentrations and tissue distribution profiles among species. Flies exhibit differences in transport, metabolism, and toxicokinetics compared to mammalian systems. Although there seems to be a strong correlation of toxicity between the two species, some innocuous drugs in flies may have a significant potential to be toxic to human and vice versa (Rand, 2010). Therefore, while dose-response data derived from studies in flies is unlikely to be directly translatable when determining the therapeutic window for use in mammals, these data can provide important information about the efficacy of a drug candidate, such as inhibiting protein aggregation within a specific tissue type *in vivo*. Notwithstanding the challenges highlighted here, drug screens in flies often exemplify the ultimate high-risk, high-reward experiment: positive hits might offer fruitful avenues for exploration of potential new drug candidates, while negative results rarely offer clues to understand why the compound failed (issues with delivery, efficacy, dosage, metabolism, BBB permeability, turnover, stability—and many others—are possible). Despite some shortcomings, Drosophila models effectively reproduce aspects of many human diseases and as such, are effective, yet simple models. The underlying conservation of genes, intrinsic cellular mechanisms and signaling pathways preserved between humans and flies are substantial enough to endorse the use of Drosophila in studying NDs.

## The Therapeutic Potential of Using *D. melanogaster* for Target Engagement- A Swarm of Options

NDs have pervasive effects from the subcellular level to the whole organism, therefore it is important that specific hallmarks of a disease are accurately represented in the animal model. Three general domains of validity for animal models of human disease include face validity, construct validity, and predictive validity (Nestler and Hyman, 2010; Narayanan and Rothenfluh, 2016). The better a model replicates the anatomical, biochemical, neuropathological, or behavioral characteristics of the human disease increases face validity of the model (Nestler and Hyman, 2010; Narayanan and Rothenfluh, 2016). Construct validity involves capturing the true cause involved in the disease progression (Nestler and Hyman, 2010). Predictive validity is how, if at all, the model responds to a treatment and how the model can be used to predict how humans would react to that treatment (Nestler and Hyman, 2010; Narayanan and Rothenfluh, 2016). The most ideal models would have each kind of validity, but because of the complex nature of NDs, creating these ideal models is difficult (Narayanan and Rothenfluh, 2016). When screening compounds for therapeutic effects in the domain of predictive validity, changes on multiple levels must be examined to assess potential therapeutic value. There are three major categories of therapeutic evaluation methods in *D. melanogaster* models of NDs based on major characteristics of neurodegeneration: morphological analysis, behavioral analysis, and biomolecular analysis. When Iijima et al. (2004) characterized their *D. melanogaster* model of AD, they studied effects of Aβ42 expression on morphology, behavior, and biomolecular attributes by examining nervous system tissue, survival, olfactory learning, and climbing and found the flies showed the hallmarks of AD: amyloid deposits, age-dependent learning deficits, and extensive neurodegeneration (Iijima et al., 2004). The same types of methods employed by Iijima et al. can be used to evaluate changes in behavior, physiology, and morphology resulting from treatment with a compound of interest.

Modifier screens, a current standard method, are used to assess toxicity of a disease-linked gene product and are often used in the development of *D. melanogaster* disease models. Typically, when a toxic product is targeted to non-essential tissues, such as the fly retinal cells, it produces an easily recognizable morphology: the rough eye phenotype (REP). As part of the classical modifier screen, the REP can be observed using light microscopy or scanning electron microscopy, with the latter being higher resolution (Trotter et al., 2017). One major advantage of these screens is the correlation between REP severity and degree of cell loss and thus indicates neurotoxicity of the gene product of interest. Additionally, these screens have a high throughput and can reveal information about epistatic interactions, or interactions between multiple genes that contribute to neurotoxicity (Lenz et al., 2013). The REP has been used effectively for screening in several neurodegenerative disease pathways including Alzheimer's disease (tau and Aβ), Parkinson's disease, polyglutamine diseases (poly Q and ataxin-1), and motor neuron disease (Lenz et al., 2013; Shulman et al., 2014; Hannan et al., 2016). Recently, an unbiased large-scale forward genetics screen of the entire fly X-chromosome (representing 15% of the fly genome) was carried out to identify essential genes that cause neurodegeneration in the fly visual system (Deal and Yamamoto, 2018). Previously, representative images of REP were used to show the effect of modification, but now, REP can be quantified using specialized analysis software. Iyer et al. (2016) developed a novel method of computation using Flynotyper software to quantify REP (Iyer et al., 2016). Other methods indicative of underlying eye phenotypes employ semi-thin sectioning of the eye to conduct morphological analysis of its cells (Jenny, 2011). Histological analysis, although lower throughput, can also be used to evaluate neurodegeneration and cell death and to verify REP data (Lenz et al., 2013). With respect to drug screening, however, REP is difficult to use because compound ingestion stops during puparium formation and the pupal stage. This stage of development is marked with high metabolic activity, so any drug previously ingested prior to puparium formation quickly loses efficacy (Hirth, 2010). Despite this complication, some studies have successfully used REP as a screening tool for therapeutics. Singh et al. (2014) used scanning electron microscopy (SEM) to evaluate the rescue of REP by a flavonoid-derived compound in a *D. melanogaster* model of AD expressing Aβ42 in the eye (Singh et al., 2014).

In addition to studying REP, other microscopy methods are used for morphological analysis of fly tissue to assess therapeutic effects of a compound. Transmission electron microscopy (TEM) has been applied to studying the toxicity of Aβ42, tau, and α-synuclein within structures of the fly eye and could be applied to analyze other neurotoxic phenomenon (e.g., synaptic changes, neuron loss) within the fly CNS (Chouhan et al., 2016). TEM has also been used to show neuroprotective effects of pomalidomide in a *D. melanogaster* model of PD and protection of mitochondria by Withania somnifera extract in a *D. melanogaster* model of ALS (De Rose et al., 2017; Casu et al., 2020). SEM examination of eye phenotypes can also be used to assess drug effects, as was the case with the study done by Singh et al. (2014), where adult flies expressing Aβ42 in the eye were raised on media containing a flavonoid derivative compound. Scoring of eye phenotypes using SEM showed rescue of severe and mild eye phenotypes and demonstrated potential therapeutic value of the compound (Singh et al., 2014). Similarly, using fluorescent microscopy, individual axon degeneration can be analyzed via the procedure outlined by Brace and DiAntonio (2020). Axon degeneration is induced in larva with the Pinch Assay or *Ex Vivo* Prep, where fluorescent staining and examination of individual axons allows for quantification of degeneration. This procedure is easily applicable to drug screens as a compound can easily be added to the medium to study the effects on axon regeneration (Brace and DiAntonio, 2020).

A major feature of NDs is reduced longevity. Lifespan experiments using fly models are useful for evaluating the efficacy of compounds as part of a screening process. Because the lifespan of a fly is relatively short, these experiments can be conducted at higher throughput than other animal models. The most common application is measuring changes to length of life, or survival; lifespan experiments can also be used to assess effects of dietary, genetic, and pharmacological interventions on survival (Piper and Partridge, 2016). Kong et al. (2016) used *D. melanogaster* models of AD to screen the compound quercetin and found rescue of survival (Kong et al., 2016). Joardar et al. (2015) conducted two survival experiments using pioglitazone in two *D. melanogaster* models of ALS and found no toxic effects of the compound on survival (Joardar et al., 2015). De Rose et al. (2017) found Withania somnifera (Wse) or Mucuna pruriens (Mpe) supplementation in food in both larval and adult stages significantly decreased survival of a *D. melanogaster* model of ALS (De Rose et al., 2017). Additionally, factors such as oxidative stress and inflammation can affect lifespan, so compounds targeting such ancillary pathways can also be evaluated for therapeutic potential using lifespan experiments (He and Jasper, 2014).

Olfactory assays are used to observe changes mainly in learning and memory, but can also be used to study vision, mechanoreception, hearing, and chemoreception (Simonnet et al., 2014). Such assays can be conducted in both adults and larvae using classical conditioning methods (Scherer et al., 2003; Malik and Hodge, 2014). T-mazes, Y-mazes, trap assays, four-field arenas and wind-tunnels are some methods used to assess olfactory function in flies (Alcorta and Rubio, 1989; Helfand and Carlson, 1989; Woodard et al., 1989; Budick and Dickinson, 2006; Faucher et al., 2006; Malik and Hodge, 2014; Simonnet et al., 2014). These assays examine both decline in and rescue of olfactory function. Age-related olfactory decline in flies was rescued by protecting against oxidative stress when flies were tested in T-maze olfactory assays (Hussain et al., 2018). Some *D. melanogaster* models of PD are able to mimic loss of olfaction seen in patients (Poddighe et al., 2013). Olfactory conditioning has been used to show rescue in AD-associated memory deficits induced by tau pathology and could be used to validate therapeutic potential of candidate compounds in a similar application (Higham et al., 2019).

Motor function is also greatly affected in patients with NDs and can be used as a proxy of neurodegeneration in fruit flies. Assaying motor function in flies can be done at early larval stages and through adulthood. Self-righting behavior in *D. melanogaster* larvae, also called larval turning, assesses locomotor function and can be performed in all larval stages. It takes advantage of the innate self-righting behavior conserved across most organisms (Issa et al., 2019). In some *D. melanogaster* models of NDs, disease phenotypes can appear as early as during larval stages, therefore studying these models at early time-points can provide significant insight (Jakubowski et al., 2012). Like other behavioral experiments, larval turning has been used to assess therapeutic potential of compounds in *D. melanogaster* models of ALS. Joardar et al. (2015) found that pioglitazone rescues larval turning time in two ALS models (Joardar et al., 2015). François-Moutal et al. (2019) showed improvement in larval turning time with rTRD01 treatment in a *D. melanogaster* model of ALS (François-Moutal et al., 2019). Critically, screening in larval stages is sometimes necessary as some *D. melanogaster* models of NDs show lethality before adulthood (Jakubowski et al., 2012).

In adult flies, negative geotaxis is an innate escape behavior in which flies reliably climb upwards after being tapped to the bottom of a container (Linderman et al., 2012). Negative geotaxis assays measure either the distance a fly climbs in a set time, the length of time it takes a fly to climb a set distance, or out of a group of flies, how many have climbed above a certain threshold over a set time (Linderman et al., 2012; Mollasalehi et al., 2020). Negative geotaxis assays are a way to validate creation of disease models by replicating deterioration of motor function as well to screen therapeutic compounds. In a study by Kong et al. (2016), *D. melanogaster* models of AD treated with quercetin showed rescue of climbing ability (Kong et al., 2016). Another study by Johnson et al. (2018) found rescue of negative geotaxis behavior in induced *D. melanogaster* models of PD treated with Mucuna pruriens seed extract (Johnson et al., 2018). The Rapid Iterative Negative Geotaxis assay (RING) is a version of a negative geotaxis assay. Instead of longer testing times, the RING assay involves short testing intervals of a few seconds for a higher throughput method compared to traditional single fly observations (Gargano et al., 2005). Like negative geotaxis assays, RING assays can be used for both genetic screens and screening compounds for therapeutic value (Liu et al., 2015). The study conducted by Lin et al. (2021) found Vitamin K2 to improve climbing ability measured by RING assay in a *D. melanogaster* model of AD (Lin et al., 2021).

Automated locomotor activity assays have been developed to increase throughput using technologies such as the Drosophila Activity Monitor (DAM) system. These systems employ beams of infrared light to monitor movement as flies move around in glass tubes with food at one end. Circadian rhythms can also be studied effectively in this system because behavior in darkness can be monitored (Pfeiffenberger et al., 2010; Cichewicz and Hirsh, 2018). Additionally, longevity, social interaction, geotaxis, learning, and phototaxis are measurable using the system (TriKinetics Inc USA, 2021). In the context of drug screening with the DAM system, flies feed freely from media containing a small compound of interest throughout the recording period (TriKinetics Inc USA, 2021). Qurashi et al. (2012) used the DAM system to assess therapeutic potential of 11 small molecules in a *D. melanogaster* model of fragile X syndrome. They found two compounds significantly rescued mean locomotor activity in flies expressing rCGG repeats (Qurashi et al., 2012).

Another automated activity system is the Frustrated Total Internal Reflection (FTIR)-based Imaging Method (FIM). FIM uses light refraction principles to create high-contrast images that can be used to track locomotion. When larvae are placed in the arena, their refractive index is higher than the transparent agar, so light enters the larval body, resulting in a detailed image of a black background and bright white larva. In addition to every larval stage, FIM can also track adult locomotion using footprints from adult flies. Developed specifically for this application, the FIMTrack program uses algorithms to detect motion based on contour features of the animal and the images captured. Quantification of locomotive behavior in larvae can then be performed using FIMTrack (Risse et al., 2014, 2017). Similar to the DAM system, to use FIM for drug screens, flies are raised in media containing the compound of interest prior to testing to assess therapeutic value. Using FIM, Sousa (2019) screened Compound C in *D. melanogaster* models of ALS expressing Fused in Sarcoma (FUS), an RNA binding protein also implicated in ALS pathology. They found Compound C treatment to have moderate therapeutic effects in flies expressing FUS; the flies expressing FUS did not return to a “normal” phenotype, but showed significant improvement with Compound C treatment (Sousa, 2019).

Automated activity assays such as DAM and FIM are critical for accelerating the screening process of flies exposed to putative therapeutics. They minimize analysis time by eliminating processing and annotation of individual animals. Such systems facilitate high throughput: monitors can have many channels and multiple monitors can record behavior of hundreds of individual flies at the same time. Automated assays generate large data sets, necessitating adequate machine learning algorithms for efficient processing. For example, tremors have been observed in fly models of neurodegeneration, yet quantification of these movements has proved difficult. Fortunately, Wu et al. (2019) have developed a novel machine learning approach to evaluating neurodegeneration and tremor movements in Drosophila using leg tracking data (Wu et al., 2019). Machine learning is integral to the relevance of automated activity assays and higher throughput of large-scale screening of therapeutic compounds.

Evaluation of small molecules and other therapeutics on a biomolecular scale is integral to the drug discovery process. Fortunately, many analytical methods can be used with *D. melanogaster* models of NDs to validate physiological effects of a compound (Pandey and Nichols, 2011). With each method, quantification and statistical analysis are integral to the therapeutic screening process by providing a stronger argument of therapeutic potential of a compound compared to representative images alone.

Fluorescence microscopy has been used extensively with *D. melanogaster* from studying cellular processes to screening therapeutic compounds, and offers a significant advantage for drug discovery studies of NDs. Labeling and visualizing cellular structures and molecules of interest enables quantification of ND hallmarks such as protein localization/aggregation and statistical analysis to evaluate therapeutic value (Rosales-Nieves et al., 2006; Daniels et al., 2008). Proteins implicated in ND pathology labeled with fluorescent tags aid in studying pathological effects, such as GFP-Aβ42, RFP-Huntingtin, tetracysteine-tagged α synuclein, and tetracysteine-tagged TDP-43 (Roberti et al., 2007; Ng et al., 2019; Bolus et al., 2020; Yeates et al., 2020). Immunostaining combined with annotated confocal microscopy can be used to quantify aggregation of specific proteins within whole tissue. This approach is valuable when evaluating small molecules that can either reduce or enhance protein expression. For example, Liu et al. used the combination of immunostaining and confocal microscopy to show ginseng total protein (GTP) supplementation in the fly diet suppresses neurodegeneration in a *D. melanogaster* model of PD (Liu et al., 2020).

Fluorescent labeling can also be used to detect abnormal cells. Astrocyte-related neuropathology has become an area of interest recently. In AD, astrocytes near Aβ plaques become reactive and cause downstream effects such as inhibiting neuronal activity and impairing memory abilities in AD patients. These reactive astrocytes can be selectively detected with fluorescent antibodies against glial fibrillary acidic protein (GFAP), a calcium-binding protein S100 beta, excitatory amino acid transporters (EAAT1/2), and aldehyde dehydrogenase-1 family (ALDH1L1). Similarly, Sulforhodamine 101 (SR101) shows strong fluorescence when labeling reactive astrocytes, but is not ideal for *in vivo* use (Jun et al., 2019).

Additionally, there are a number of fluorescent markers available for studying the aggregates associated with NDs. The fluorescent dye thioflavin T (ThT) was used initially to stain amyloid fibrils in histological samples. When the dye binds to the architecture of amyloid fibrils, the excitation and emission wavelengths shift, resulting in a strong increase in fluorescent emission. Thus, ThT detects amyloid fibril formation, as well as aggregation of amyloid-β and α-synuclein *in vitro* in aggregation assays (Wördehoff and Hoyer, 2018). ThT was employed to show that amentoflavone-type biflavonoids disrupt hydrogen bonding in the aggregate structure of Aβ and thus disaggregate Aβ fibrils (Windsor et al., 2021). Higher-throughput assays such as real-time quaking induced conversion assay (RT-QulC) also employ ThT to evaluate aggregation of prion proteins (Schmitz et al., 2016; Favole et al., 2019). Thioflavin S (ThS) is similar to ThT as it binds to amyloid fibrils and fluorescence is enhanced, but binding does not result in a spectral shift like ThT (LeVine, 1999). Congo Red (CR) is another dye traditionally used for the identification of amyloids *in vitro* and in tissue sections. However, CR staining for diagnostic use has a high rate of false positives and false negatives when injected into patients but is commonly used to evaluate tissue samples and biopsies (Yakupova et al., 2019). Despite the utility of these dyes, there are some important limitations. ThT and other conventional aggregation markers only show the overall transition from soluble protein to the aggregated form. There is ongoing research to develop better biomarkers and fluorescent small molecules to use for diagnostic or research purposes to detect pathogenic protein aggregation. A comprehensive review of the development of new probes for AD can be found by Jun et al. (2019). Bis(triphenylphosphonium) tetraphenylethene (TPE-TPP), a different dye, can identify three distinct aggregation intermediates of Aβ (Das et al., 2020). Dipolar dyes and derivatives are being developed to detect misfolded amyloid-β species and monoamine oxidases (MAOs). Detection of phosphorylated tau with fluorescent dyes has proved difficult as selectivity for p-tau over Aβ plaques has not yet been reliably achieved. However, recent studies using aryl-quinoline derivatives, phenyldiazenyl-benzothiazole (PDB) and styryl-benzimidazole (SBIM) scaffolds, thiohydantoin based p-tau probe (TH2), and 18F-T807 appear promising for selective detection of p-tau (Jun et al., 2019). Neurodegeneration can also involve disruption of metal ion homeostasis, so detection of high levels of heavy metal ions is useful for the study of NDs. Iminopyridyl chelates and dual probes based on iminopyridyl chelates can be used to detect metal-induced Aβ aggregates *in vitro* as well as in human neuroblastoma cells (Jun et al., 2019).

Fluorescent technology can also be used to control cell functions with optogenetic tools. Expression of light-sensitive proteins can be manipulated with laser light. Neuronal activity has been regulated using light-gated variants of channelrhodopsin-2 (Tyrer et al., 2000; Dunst and Tomancak, 2019). Optogenetics have even been applied to the creation of a new *D. melanogaster* model of AD where amyloid β oligomerizes when exposed to blue light (Lim et al., 2020). Cell lysate prepared from *D. melanogaster* tissue or cell cultures are particularly useful for studying protein biochemistry and are routinely used for co-immunoprecipitation (co-IP) experiments, Western blot analysis, and enzyme-linked immunosorbent assay (ELISA) (Emery, 2007; Wang et al., 2019). An additional tool to complement other regularly used protein-protein interaction assays is Proximity Ligation Assay (PLA). PLA uses primary antibodies against the two proteins of interest using standard immunohistochemical procedures and is capable of determining within a tissue the subcellular localization of endogenous protein interactors that are in close proximity and likely forming a complex (Wang et al., 2015). In the context of ND drug discovery, co-IP experiments are performed to assess and quantify drug-induced changes in protein-complex formation where the compound of interest targets protein interactions (Nikolsky et al., 2005). Tsuburaya et al. (2018) performed *in vitro* co-IP assays to identify a lead compound targeting SOD1mut-Derlin-1 interactions implicated in ALS as triggering motoneuron death (Tsuburaya et al., 2018). Western blot analysis can be performed from cell or tissue homogenates to evaluate the therapeutic potential of a compound that affects protein interactions or expression levels of a gene-product of interest (Trotter et al., 2017). Chouhan et al. (2016) used western blot analysis to quantify levels of tau and α-synuclein in respective *D. melanogaster* lines (Chouhan et al., 2016). These authors also used ELISA to quantify total Aβ42 expression in a *D. melanogaster* model of AD (Chouhan et al., 2016). These applications are especially useful for ND models as many involve pathogenic protein aggregation and quantification of protein expression changes may identify therapeutic effects on a molecular level. Assays requiring milligram or higher amounts of protein simply require harvesting large numbers of flies to get adequate amounts of lysate for the specific method. Some of the more recent advances include genome-wide approaches for the analysis of protein localization in Drosophila and proteomic profiling (Sarov et al., 2016; Du et al., 2018), both boosting systematic analysis of protein expression and localization in various cellular and developmental contexts and providing a basis for further mechanistic studies of targeted protein.

**Figure 2:**
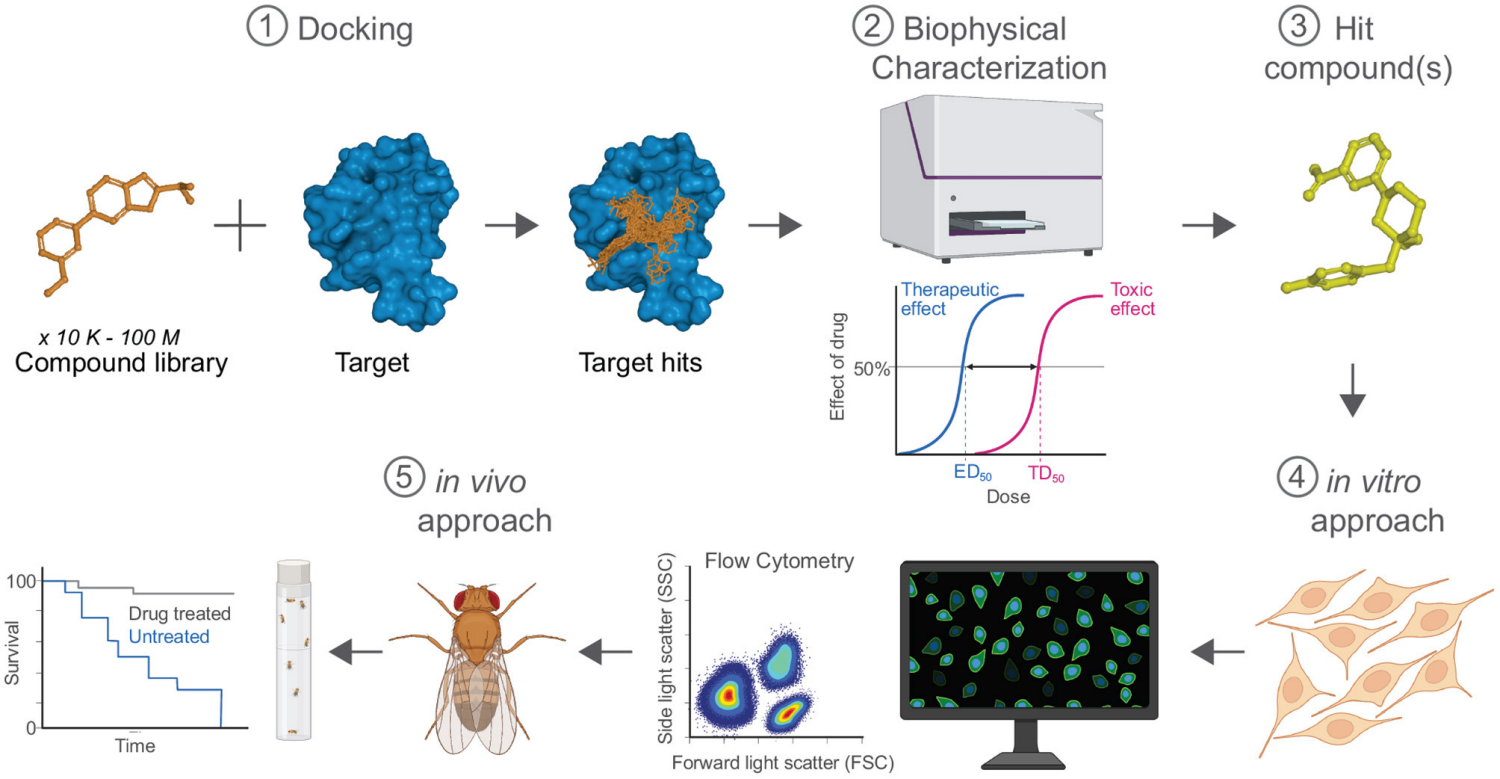
Target-directed approach in the drug discovery pipeline for ND therapies. Building upon on the individual strengths of multiple techniques, this integrated approach synergistically accelerates the identification of compounds for clinical development. *In silico* docking (1) is used to identify potential therapeutic compounds using computer models and mathematical methods. The *in-silico* leads are further characterized using biophysical methods (2) to assess target:candidate binding, thermodynamics, and pharmacokinetic/pharmacodynamic parameters. Hit compounds (3) are identified and subjected to *in vitro* screening protocols (4) using the appropriate cell culture conditions, e.g., NSC and neurons from patient-derived tissue samples that are subsequently analyzed using flow cytometry. To assess pre-clinical effects of hit compounds, flies (5) serve as an *in vivo* animal model of disease. Created with BioRender.com.

The more traditional workflow of drug discovery is not sufficient to keep up with the increasing need for better therapeutics. Modern high-throughput technologies significantly reduce preclinical screening of compounds of interest (Figure 2). To improve efficiency of compound identification and screening, some investigations have implemented a target-direct approach to the drug discovery pipeline for ND therapies. By using the information about the pathogenesis of a disease, a drug target is used to guide small molecule identification. Use of ever-improving modern approaches such as computer modeling and *in silico* compound libraries has already been shown to increase speed of preclinical identification of therapeutic compounds (Aldewachi et al., 2021). *In silico* docking experiments are performed using a compound library and positive hits are further investigated using biophysical and biochemical experiments. For *in vivo* validation, physiological and biomolecular tools available in flies allows validated hit candidate molecules to be expediently tested in the relevant ND model system (Papanikolopoulou et al., 2019).

A real-world example illustrating the power of Drosophila in a modern high-throughput pipeline was recently demonstrated using three transgenic ALS models. In ALS, TAR DNA-binding protein 43 (TDP-43) forms aggregates in motor neurons that negatively affect axon formation and could contribute to neurodegeneration (Oberstadt et al., 2018). Two key structural domains in TDP-43 that contribute to ALS pathogenesis were targeted for therapeutic screening: the RNA Recognition Motif 1 (RRM1) and N-terminal domain (NTD). RRM1 is involved in RNA binding and could cause RNA dysregulation in the diseased state. The NTD was proposed to dimerize and promote aggregation of TDP-43 (François-Moutal et al., 2019). Using this information, François-Moutal et al. (2019) and Mollasalehi et al. (2020) hypothesized that targeting the RRM1 and N-terminal domains of TDP-43, respectively, could affect disease pathogenesis. *In silico* docking of TDP-43 with a compound library targeting the RRM1 and NTD domains separately identified compounds of interest that could disrupt the described interactions occurring in RRM1 and NTD. From these hits, biophysical and biochemical experiments were performed to screen each hit, and compounds identified by predicted binding affinity were therefore validated (François-Moutal et al., 2019; Mollasalehi et al., 2020).

The study performed by François-Moutal et al. (2019) identified rTRD01, a compound targeting the RRM1 domain of TDP-43 that disrupted interactions between RRM1 and disease-linked nucleic acids without interfering with TDP-43 binding to normal nucleic acids. rTRD01 interacting with the RRM1 domain of TDP-43 was confirmed through STD NMR and characterized through microscale thermophoresis (MST), 15N−1H heteronuclear single quantum correlation spectroscopy (HSQC) and amplified luminescent proximity homogeneous alpha assay (ALPHA). *In vitro* testing in two *D. melanogaster* models of ALS expressing either TDP-43WT or TDP-43G298S via a GAL4-UAS expression system showed improvement in larval turning time with rTRD01 treatment (François-Moutal et al., 2019). Mollasalehi et al. (2020) used a similar framework to identify nTRD22, another compound targeting the NTD of TDP-43 that has an indirect effect on the RNA-binding domain. In this study, fluorescent images of cortical neuron cultures were also used to characterize effects of nTRD22. It was also shown that nTRD22 has neuroprotective properties in a fly model of ALS. Moreover, climbing assays using a *D. melanogaster* model of ALS overexpressing TDP-43WT showed increased climbing behavior when the flies were treated with nTRD22 (Mollasalehi et al., 2020).

## Conclusion

As the human population ages, the prevalence of aging diseases increases, placing an ever-increasing burden on healthcare systems around the world. Neurodegenerative diseases (NDs) stand out due to the lack of effective therapies. The burden of these diseases is expected to grow at an increasing rate unless more effective therapies are developed, or cures are discovered. Critical to the search for better therapeutics and cures for NDs is the use of animal models of human diseases to screen compounds for therapeutic potential. Among available models, *Drosophila melanogaster* (fruit fly) models of human NDs provide a more efficient means of screening compounds. These animals have high genetic similarity to humans and ~75% of human disease-related genes have homologs in *D. melanogaster*. Because NDs are aging diseases, studying therapeutic effects as an animal ages is critical to screening. Fruit flies have a lifespan of about 50–80 days, so less time needs to be invested to collect advanced-age data timepoints. Physiologically, fruit flies and vertebrates have many structural and functional similarities in the brain and nervous system, allowing for a high level of translatability of therapeutic effects. Compound screening can be done in a number of ways, but the most valuable and reliable information is collected when including multiple screening methods. Using morphological, behavioral, and biomolecular analyses together can provide strong evidence for therapeutic value if improvement is observed across different disease hallmarks.

Efficient drug discovery pipelines are essential to lessen the burden of NDs on health care systems, patients, and care givers. Fruit fly models of NDs are situated in a “sweet spot” of possessing high similarity to complex disease pathologies in humans with the simplicity of applications employing high throughput screening methods. Although using fruit flies to model human diseases is nothing new, using twenty-first century technologies and novel drug discovery methods in conjunction with ever-improving disease models is integral to finding more effective therapeutics and with hope, durable cures.

## Author Contributions

JT and HW wrote the manuscript. JT designed the review and elaborated the figures. RE created Table 2. MS oversaw the creation of Table 2 and Figure 1, wrote several sections, and edited the manuscript. MK oversaw the creation of the manuscript and edited it. All authors contributed to the article and approved the submitted version.

## Funding

FRCE grant (to MS) and Summer Scholar Award (to RE) from the Office of Research and Graduate Studies at Central Michigan University.

## Conflict of Interest

The authors declare that the research was conducted in the absence of any commercial or financial relationships that could be construed as a potential conflict of interest.

## Publisher's Note

All claims expressed in this article are solely those of the authors and do not necessarily represent those of their affiliated organizations, or those of the publisher, the editors and the reviewers. Any product that may be evaluated in this article, or claim that may be made by its manufacturer, is not guaranteed or endorsed by the publisher.
